# Supplementary material for: Pregnancy-acquired memory CD4+ regulatory T cells improve pregnancy outcome in mice
Source: Nat Commun. 2025 Jul 15;16:6522. doi: 10.1038/s41467-025-61572-w (PMC12263984; doi:10.1038/s41467-025-61572-w)
Supplement: Supplementary file 1 — Supplementary Information [file 41467_2025_61572_MOESM1_ESM.pdf]

SUPPLEMENTAL INFORMATION

Pregnancy-acquired memory CD4+ regulatory T cells improve pregnancy outcome in mice

Kristin Thiele[1,2*], Christopher Urbschat[1,2], Julia Isabel Amambay Riquelme[1], Lisa Sophie Ahrendt[1], Ronja Wöhrle[1], Steven Schepanski[1,3], Judith Joana Eckert[1,4], Etienne Becht[5], Minyue Qi[6], Malik Alawi[6], Martin Becker[7], Nicola Gagliani[2,8,9], Hans-Willi Mittrücker[10], Anke Diemert[11,12], Petra Clara Arck[1,2,12*]

Affiliations

[1] Division of Experimental Feto-Maternal Medicine, Department of Obstetrics and Fetal Medicine, University Medical Center Hamburg-Eppendorf, Hamburg, Germany.

[2] Hamburg Center for Translational Immunology, University Medical Center Hamburg-Eppendorf, Hamburg, Germany.

[3] Institute of Developmental Neurophysiology, Center for Molecular Neurobiology Hamburg (ZMNH), University Medical Center Hamburg-Eppendorf, Hamburg, Germany.

[4] Human Development and Health, Southampton General Hospital, University of Southampton, Southampton, UK.

[5] Vaccine and Infectious Disease Division, Fred Hutchinson Cancer Research Center, Seattle, USA.

[6] Bioinformatics Core, University Medical Center Hamburg-Eppendorf, Hamburg, Germany.

[7] Chair for Intelligent Data Analytics, Institute for Visual and Analytic Computing, Department of Computer Science and Electrical Engineering, University of Rostock, Rostock, Germany.

[8] Section of Molecular Immunology and Gastroenterology, I Department of Medicine, University Medical Center Hamburg-Eppendorf, Hamburg, Germany.

[9] Department of General, Visceral and Thoracic Surgery, University Medical Center Hamburg-Eppendorf, Hamburg, Germany.

[10] Institute for Immunology, University Medical Center Hamburg-Eppendorf, Hamburg, Germany.

[11] Department of Obstetrics and Fetal Medicine, University Medical Centre Hamburg-Eppendorf, Hamburg, Germany.

[12] German Center for Child and Adolescent Health, Hamburg, Germany

* Lead contact, Correspondence: Kristin Thiele: k.thiele@uke.de
Petra Arck: p.arck@uke.de

This supplemental material includes:

Supplementary figures 1-9

Supplementary table 1

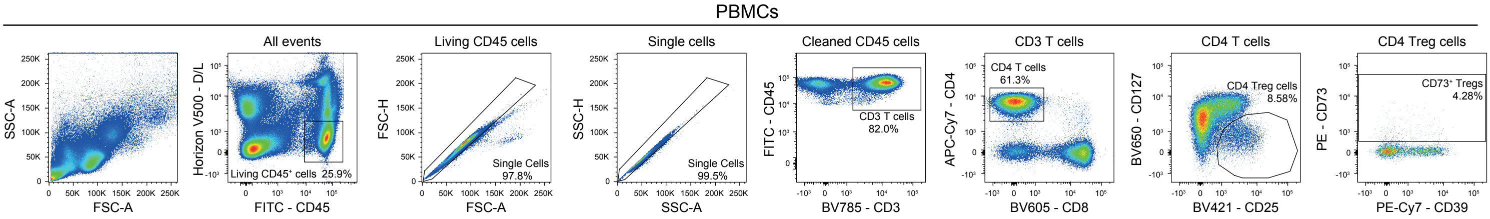

Supplementary Fig. 1: Gating strategy for flow cytometry analysis of peripheral blood mononuclear cells (PBMCs) obtained from 15 pregnant women in each trimester of their 1st and 2nd pregnancy, respectively.

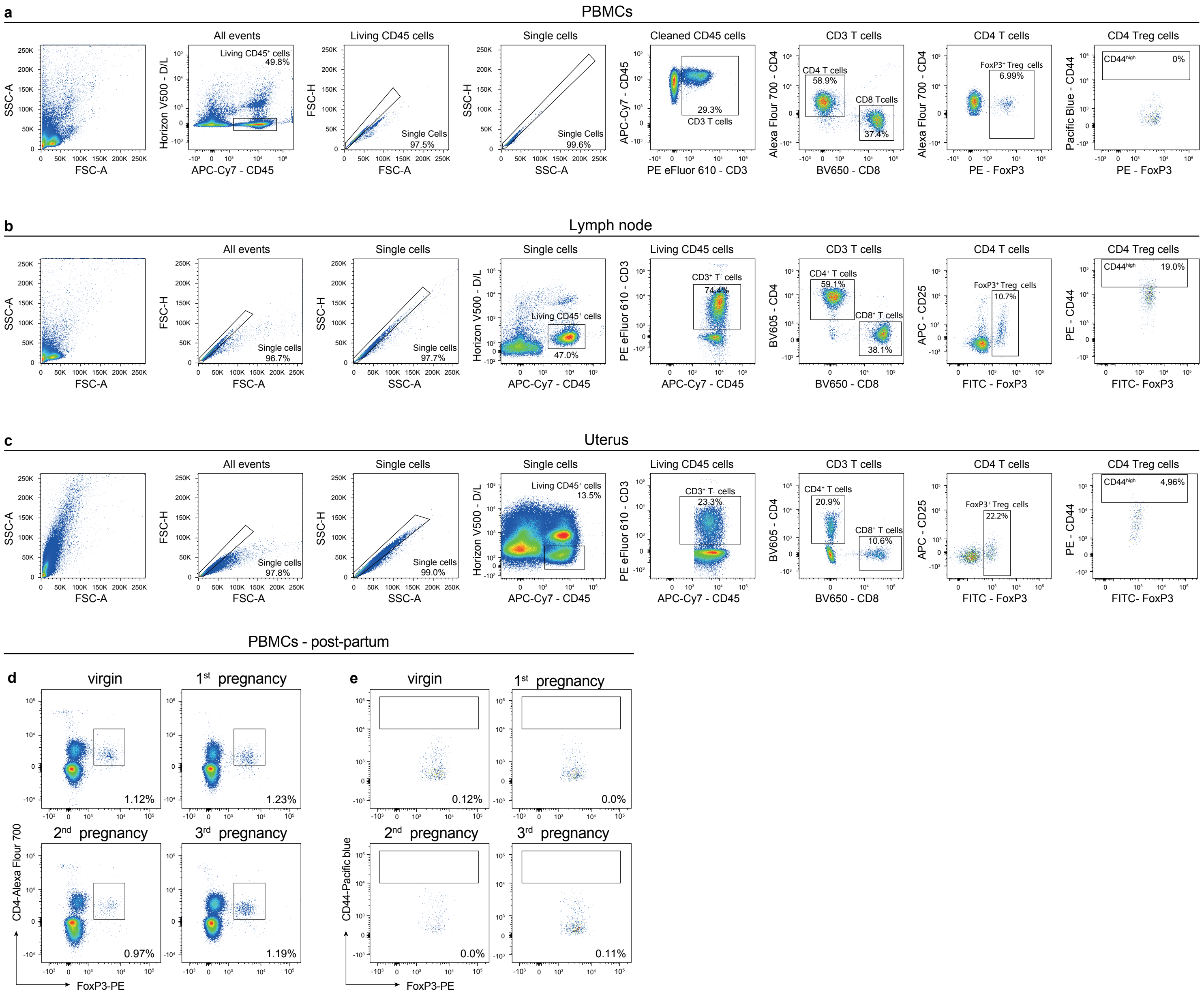

Supplementary Fig. 2: CD4⁺ regulatory T (Treg) frequencies in different murine tissues. Experimental setup: age-matched C57Bl/6J mice were allogenically mated to Balb/c males once, twice or three times, respectively, and compared to virgin mice at the age of 21 weeks. Flow cytometry analysis was performed 4 weeks after the last delivery to assess CD4⁺ Treg cell frequencies. **a-c** Gating strategy for post-partum analysis of peripheral blood mononuclear cells (PBMCs, **a**), lymph node (**b**) and uterus (**c**). **d, e** Pseudicolor plots are presented for CD4⁺ Treg cell frequencies (**d**) and their associated CD44^{high} expression (**e**) in PBMCs.

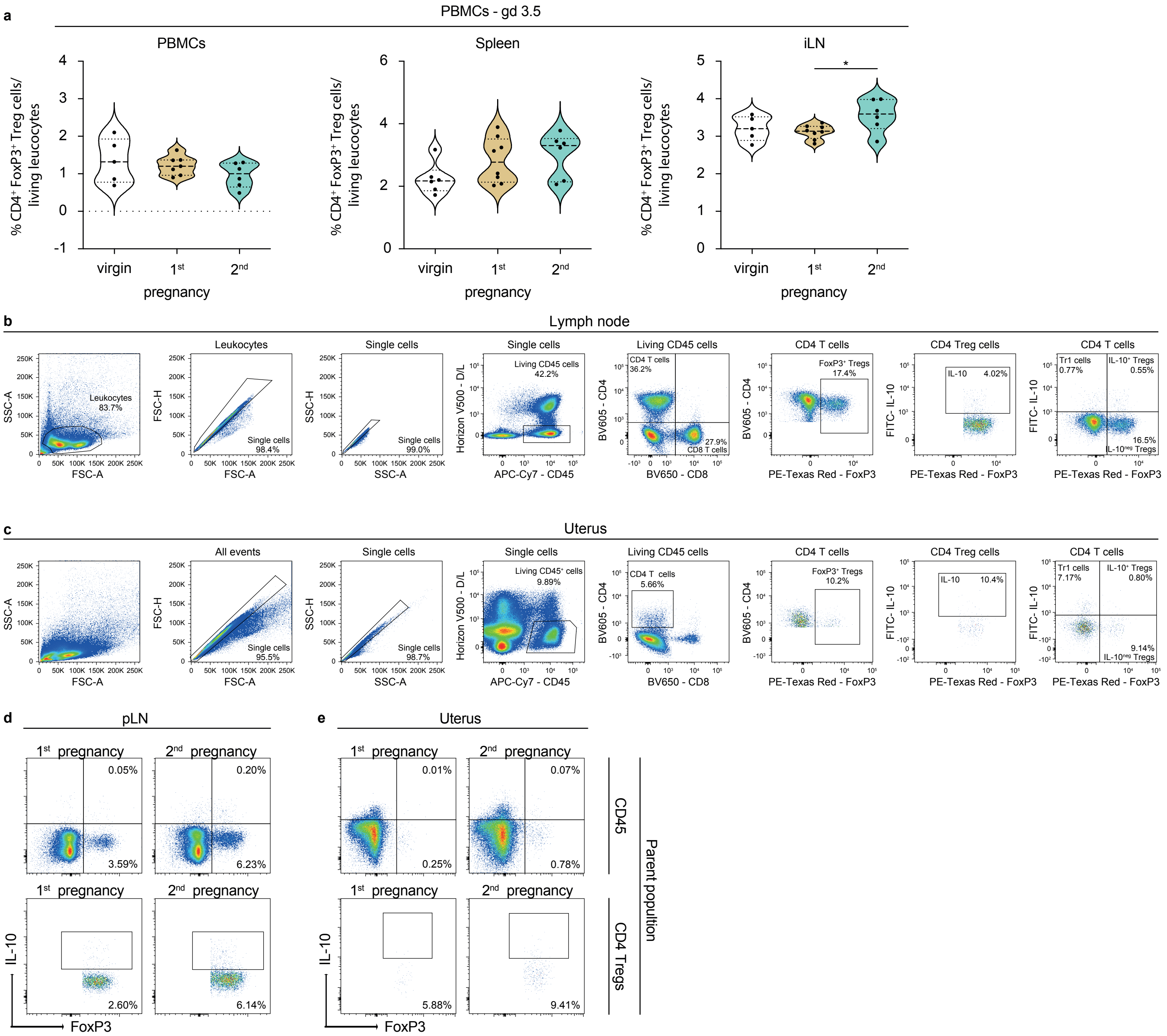

Supplementary Fig. 3: CD4$^+$ regulatory T (Treg) frequencies in different tissues during gestation. a-e Experimental setup: age-matched Fir/Tiger mice were allogenically mated to Balb/c males once or twice, respectively, and evaluated on gestational day (gd) 3.5. **a** Flow cytometry analysis included frequencies of CD4$^+$ Treg cells in peripheral blood mononuclear cells (PBMCs; virgin: n=5, 1st: n=7 and 2nd: n=6), spleen (virgin: n=6, 1st: n=8 and 2nd: n=6) and inguinal lymph node (iLN; virgin: n=5, 1st: n=8 and 2nd: n=6; 1st vs 2nd: p=0.049). Data are presented as violin plots with individual points, median and quartiles, and the statistical significance between groups was calculated using One-way-Anova (* $p < 0.05$). Source data are provided as a Source Data file. **b, c** Gating strategy for flow cytometry analysis during gestation of lymph node (**b**) and uterus (**c**). **d, e** Flow cytometry analysis included frequencies of CD4$^+$ Treg cells and associated *ex-vivo* IL-10 expression in paraaortic lymph node (pLN, **d**) and uterus (**e**). Pseudocolor plots and respective numbers presented correspond to CD45 (top) or CD4$^+$ Treg cells (bottom) as parent population.

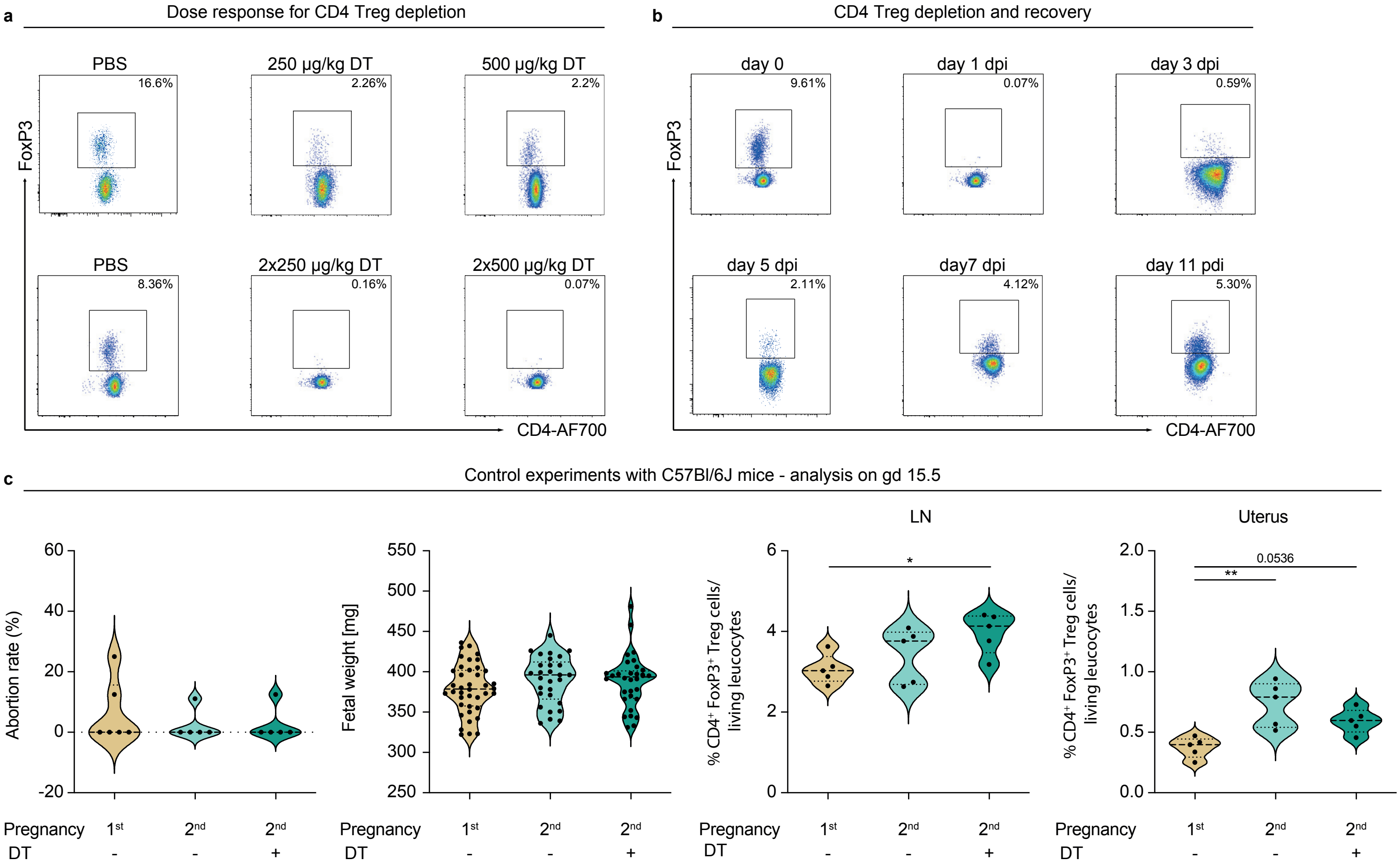

Supplementary Fig. 4: Establishment of the DEREG (DEpletion of REGulatory T cells) mouse model. a DEREG mice were injected with 250 or 500 µg/kg bodyweight diphteria toxin (DT), either once (top) or twice at an interval of 24 h (bottom). CD4^{+} Treg cell frequencies in the lymph node were detemined 24 h after the last DT injection. **b** Mice were injected twice with 500 µg/kg bodyweight DT and CD4^{+} Treg cell frequencies in the lymph node were assessed on different days post-injection (dpi). **c** In order to assess side effects of the DT injection, age-matched C57Bl/6J mice were allogenically mated to Balb/c males once or twice, respectively. Subsequently, some second pregnancy mice were injected with diphteria toxin (DT) after the first delivery and subjected to a recovery phase of 14 days before re-mating. On gestational day (gd) 15.5, abortion rate (1st: n=6, 2nd: 5 and 2nd+DT: n=5), fetal weight (1st: n=40 (6 litter), 2nd: 29 (5 litter) and 2nd+DT: n=32 (5 litter)) as well as CD4^{+} Treg cell frequencies in lymph node and uterus (1st: n=5, 2nd: 5 and 2nd+DT: n=5; LN: 1st vs 2nd+DT: p=0.0484; uterus: 1st vs 2nd: p=0.0026) were determined. Data are presented as violin plots with individual points, median and quartiles, and the statistical significance between groups was calculated using One-way-Anova (* $p < 0.05$, ** $p < 0.01$). Source data are provided as a Source Data file.

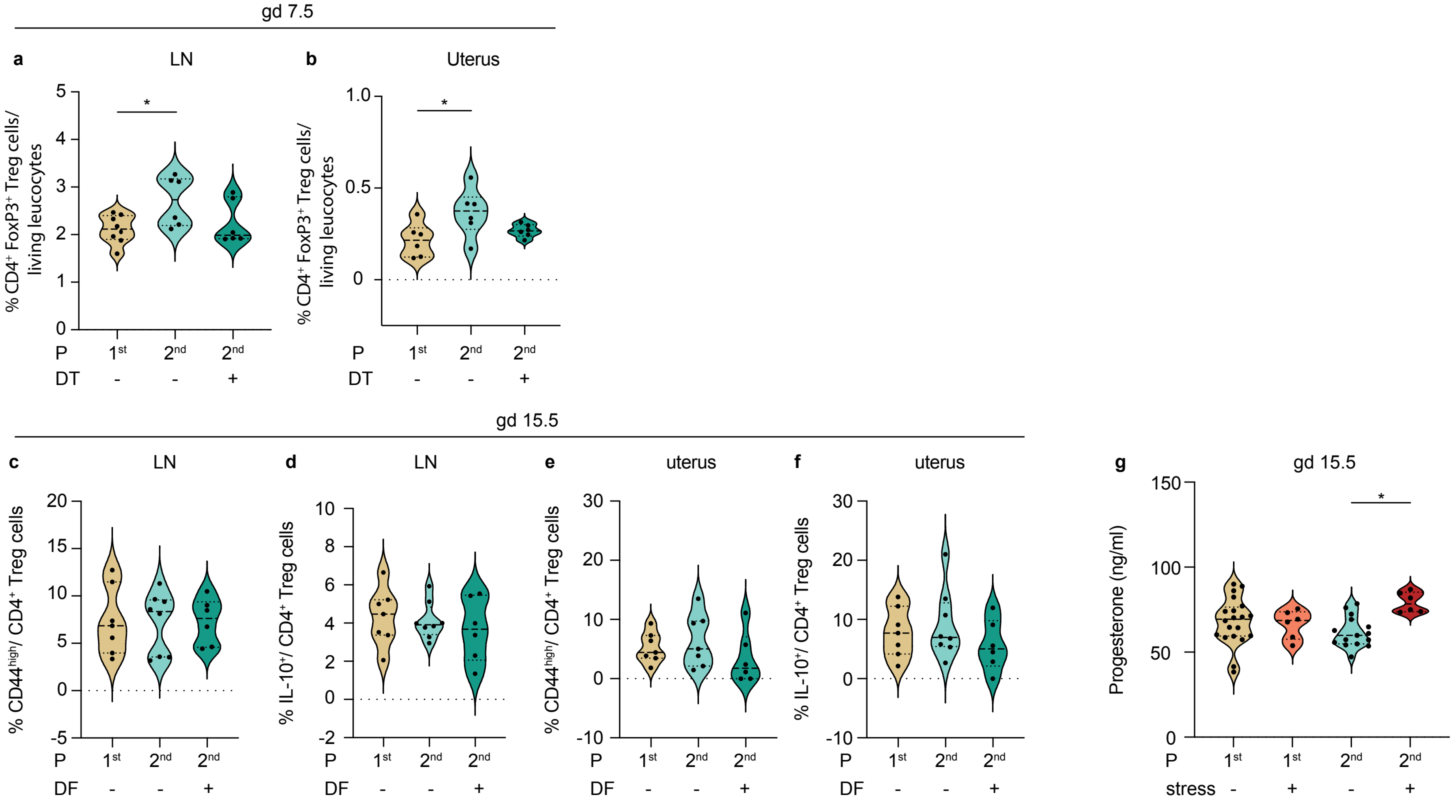

Supplementary Fig. 5: Modulation of CD4⁺ regulatory T (Treg) cell number and antigen-specificity during pregnancies. a, b Experimental setup: age-matched DEREG mice were allogenically mated to Balb/c males once or twice, respectively. Some second pregnancy mice were injected with diphteria toxin (DT) after the first delivery to deplete CD4⁺ Treg cells and subjected to a recovery phase of 14 days. On gestational day (gd) 7.5, frequencies of CD4⁺ Treg cells in (**a**) lymph node (1st: n=8, 2nd: 6 and 2nd+DT: n=6; 1st vs 2nd: p=0.0480) and (**b**) uterus (1st: n=6, 2nd: 6 and 2nd+DT: n=6; 1st vs 2nd: p=0.0325) were assessed by flow cytometry. **c-f** Experimental setup: age-matched Fir/Tiger mice were allogenically mated to Balb/c males once or twice, respectively. Additionally, some mice were mated to DBA mice for their second pregnancy (DF=different father). On gd 15.5, frequencies of CD44^{high} and IL-10 expression on CD4⁺ Treg cells in lymph node (**c, d**) and uterus (**e, f**) were assessed by flow cytometry (1st: n=7, 2nd: 8 and 2nd+DF: n=6). **g** Experimental setup: age-matched Fir/Tiger mice were allogenically mated to Balb/c males once or twice, respectively. Additionally, first and second pregnancy mice were prenatally sound-stressed mid-gestationally on gd 10.5, 12.5 and 14.5. On gd 15.5, serum progesterone levels were assessed by ELISA (1st: n=20, 1st+stress: n=6, 2nd: n=15 and 2nd+stress: n=6; 2nd vs 2nd+stress: p=0.0211). Data are presented as violin plots with individual point, median and quartiles, and the statistical significance between groups was calculated using One-way-Anova (* $p < 0.05$). Source data are provided as a Source Data file.

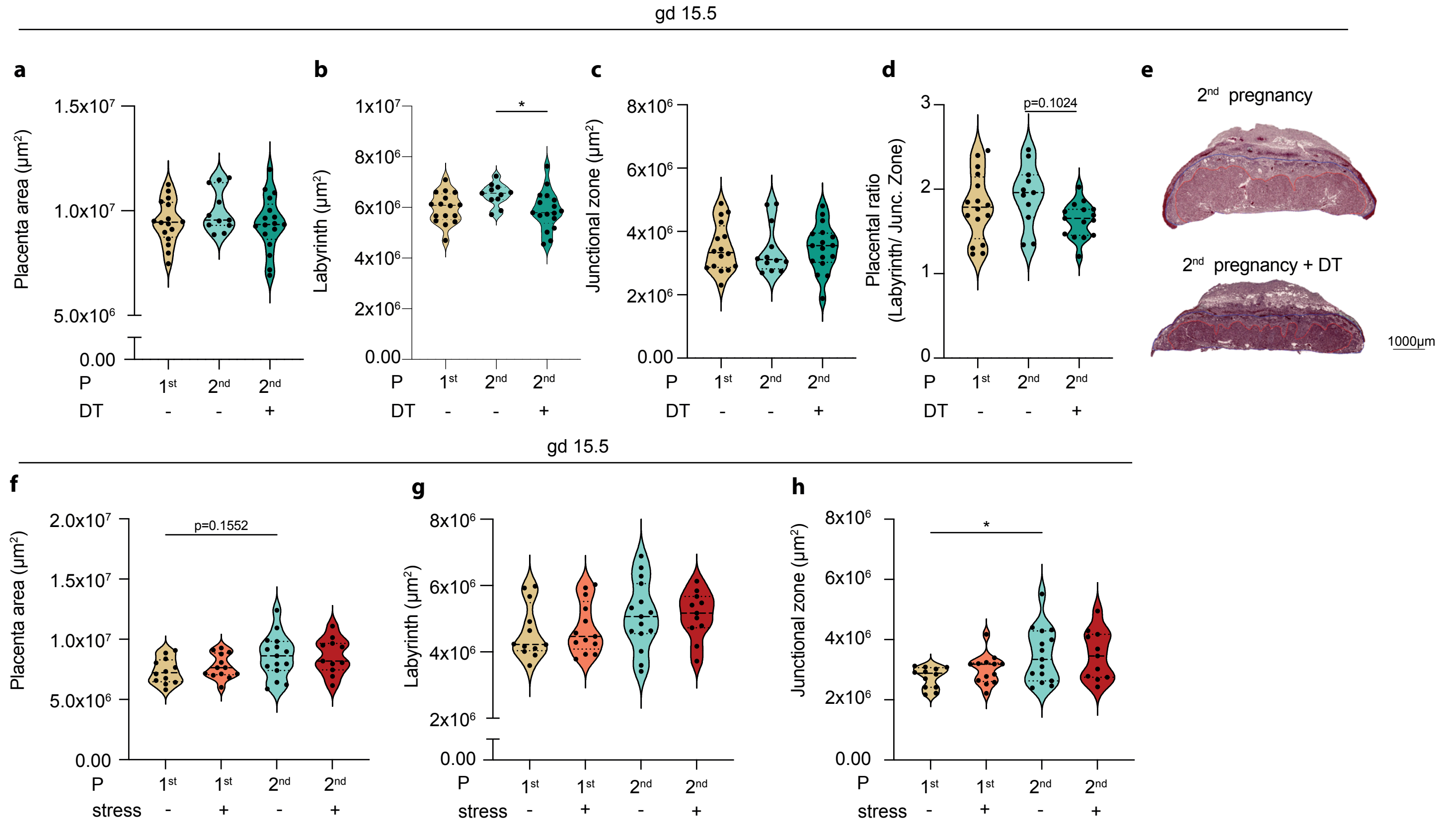

Supplementary Fig. 6: Consequences of $CD4^+$ regulatory T (Treg) cell modulation on pregnancy outcome. a-e Experimental setup: age-matched DEREG mice were allogenically mated to Balb/c males once or twice, respectively. Some second pregnancy mice were injected with diphteria toxin (DT) after the first delivery to deplete $CD4^+$ Treg cells and subjected to a recovery phase of 14 days. On gestational day (gd) 15.5, placental histomorphology was assessed including (**a**) overall placental area (1st: n=16, 2nd: n=11 and 2nd+DT: n=17), (**b**) labyrinth and (**c**) the junctional zone. (**d**) Placental ratio was calculated. (**e**) Representative photomicrographs illustrate mid-sagittal sections of placental tissue from second (top) and second pregnancy after DT administration (bottom). Black line in the picture denotes 1000 µm, red lines encircle the labyrinth, blue lines surround the junctional zone. **f-h** Experimental setup: age-matched Fir/Tiger mice were allogenically mated to Balb/c males once or twice, respectively. Additionally, first and second pregnancy mice were prenatally sound-stressed mid-gestationally on gd 10.5, 12.5 and 14.5. On gd 15.5, placental histomorphology was assessed including (**f**) overall placental area (1st: n=12, 1st+stress: n=13, 2nd: n=15 and 2nd+stress: n=11), (**g**) labyrinth and the (**h**) junctional zone (1st vs 2nd: p=0.0484). Data are presented as violin plots with individual point, median and quartiles, and the statistical significance between groups was calculated using One-way-Anova (* $p < 0.05$). Source data are provided as a Source Data file.

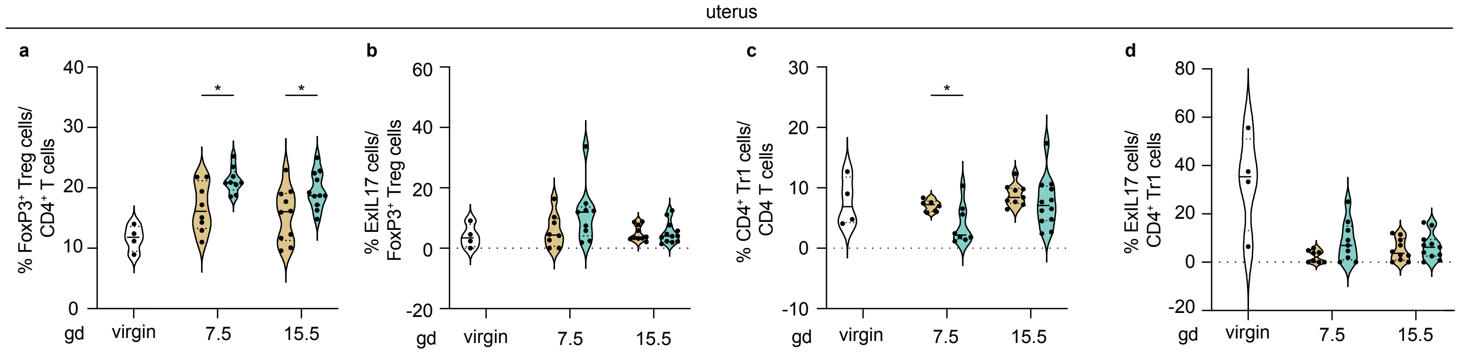

Supplementary Fig. 7: Plasticity of $CD4^+$ regulatory T (Treg) cells in first and second pregnancies. Experimental setup: age-matched Fate mice were allogenically mated to Balb/c males once or twice, respectively. **a-d** Flow cytometric analysis of uterine tissue harvested on different gestational days was performed to assess frequencies of (**a**) $CD4^+$ Treg cells (virgin: n=4, gd 7.5: 1st: n=8 and 2nd: n=9; 1st vs 2nd: p=0.010, gd 15.5: 1st: n=10 and 2nd: n=11; 1st vs 2nd: p=0.0229) along with the frequency of (**b**) exIL-17 cells within the $CD4^+$ Treg population (virgin: n=4, gd 7.5: 1st: n=8 and 2nd: n=9, gd 15.5: 1st: n=10 and 2nd: n=12) and (**c**) Type 1 regulatory T (Tr1) cell frequency (virgin: n=4, gd 7.5: 1st: n=6 and 2nd: n=8, p=0.0426, gd 15.5: 1st: n=9 and 2nd: n=12) in association with the frequency of (**d**) exIL-17 cells within the Tr1 cell compartment (virgin: n=4, gd 7.5: 1st: n=8 and 2nd: n=9, gd 15.5: 1st: n=10 and 2nd: n=11). Data are presented as violin plots with individual points, median and quartiles, and the statistical significance between first and second pregnancy was calculated using multiple unpaired t-tests (* $p < 0.05$). Source data are provided as a Source Data file.

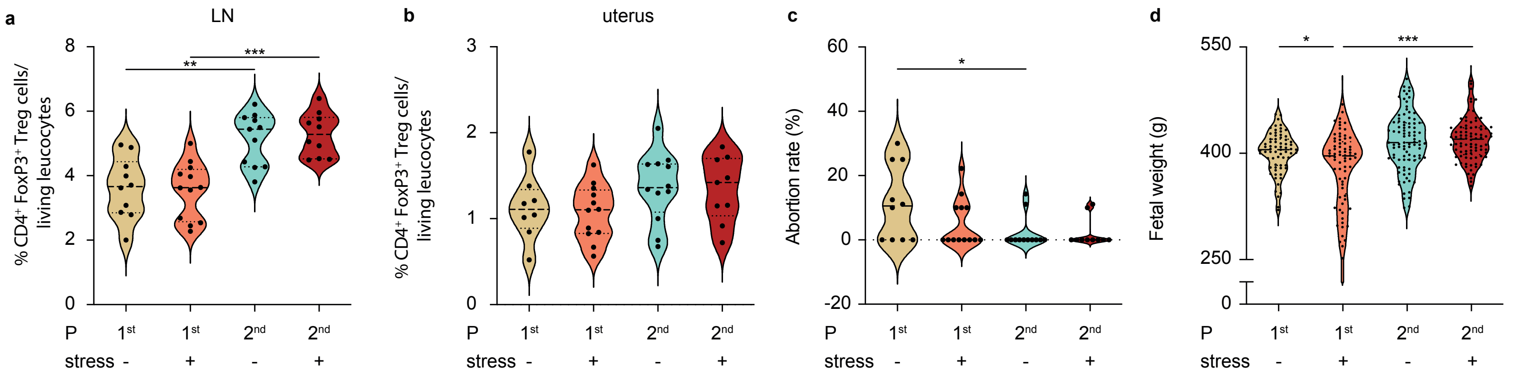

Supplementary Fig. 8: Independent confirmation of prenatal stress-induced changes in maternal immune adaptation and fetal outcome in Fate mice. a-d Experimental setup: age-matched Fate mice were allogenically mated to Balb/c males once or twice, respectively. Additionally, first and second pregnancy mice were prenatally sound-stressed mid-gestationally on gd 10.5, 12.5 and 14.5. On gd 15.5, flow cytometry analysis assessed $CD4^+$ Treg cell frequencies in (**a**) lymph node (1st: n=10, 1st+stress: n=12, 2nd: n=11 and 2nd+stress: n=10; 1st vs 2nd: p=0.0015, 1st+stress vs 2nd+stress: p=0.0002) and (**b**) uterus (1st: n=8, 1st+stress: n=12, 2nd: n=12 and 2nd+stress: n=9). Further, (**c**) abortion rate (1st: n=10, 1st+stress: n=13, 2nd: n=12 and 2nd+stress: n=10; 1st vs 2nd: p=0.0406) and (**d**) fetal weight (1st: n=68 (10 litter), 1st+stress: n=72 (12 litter), 2nd: n=91 (12 litter) and 2nd+stress: n=86 (10 litter); 1st vs 1st+stress: p=0.0107, 1st+stress vs 2nd+stress: p=<0.0001) were determined on gd 15.5 in response to prenatal stress. Data are presented as violin plots with individual point, median and quartiles, and the statistical significance between groups was calculated using One-way-Anova (* $p < 0.05$, ** $p < 0.01$, *** $p < 0.001$). Source data are provided as a Source Data file.

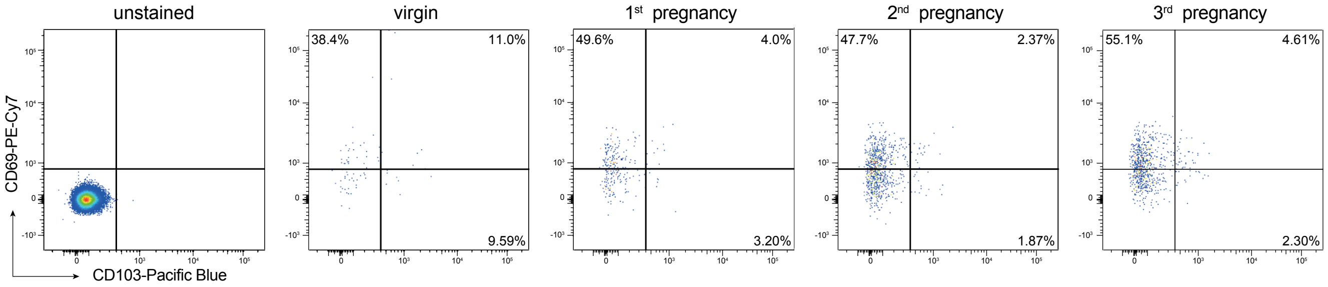

Supplementary Fig. 9: **Expression of CD69 and CD103 on uterine CD4^{+} regulatory T (Treg) cells in subsequent pregnancies:** Experimental setup: age-matched C57Bl/6J mice were allogenically mated to Balb/c males once, twice or three times, respectively, and compared to virgin mice. Flow cytometry analysis was performed 4 weeks after the last delivery at the age of 21 weeks. Number in the respective corners of the dot plots represent the frequency of single CD69^{+} or CD103^{+} expression or CD69^{+}/CD103^{+} co-expression on CD4^{+} Treg cells.

Supplementary table 1: Antibodies used in flow cytometry

Antigen/ Target	Conjugated fluorochrome	Clone	Company	Dilution	Catalog number	IDENTIFIER
**Mouse**						
CD45	APC-Cy7	30-F11	BD	1:400	422302	RRID: AB_2818986
CD3	PE-Cy7	145-2C11	Biolegend	1:200	100319	RRID:AB_312684
CD3	PE eFluor® 610	145-2C11	eBioscience	1:100	61-0031-80	RRID:AB_2574513
CD8	BV650	53-6.7	Biolegend	1:100	100741	RRID:AB_11124344
CD4	BV605	RM4-5	Biolegend	1:200	100547	RRID:AB_2563054
CD4	AF700	GK1.5	Biolegend	1:200	100430	RRID:AB_493699
CD25	APC	PC61	Biolegend	1:200	102011	RRID:AB_312860
CD25	BV605	PC61	Biolegend	1:200	102035	RRID:AB_11126977
CD44	PE	IM7	BD	1:100	553134	RRID:AB_394649
CD44	Pacific blue	IM7	Biolegend	1:100	103020	RRID:AB_493683
CD62L	BV711	MEL-14	Biolegend	1:200	104445	RRID:AB_2564215
FoxP3	FITC	FJK-16s	eBioscience	1:100	11-5773-80	RRID:AB_465242
FoxP3	PE	FJK-16s	eBioscience	1:100	12-5773-80	RRID: AB_465935
CD69	PE-Cy7	H1.2F3	BD	1:100	552879	RRID:AB_394508
CD103	Pacific Blue	2F7	Biolegend	1:100	121418	RRID:AB_2128619
CD11c	BV785	N418	Biolegend	1:100	117335	RRID:AB_2565268
CD11b	PE-Cy7	M1/70	Biolegend	1:400	101216	RRID:AB_312799
CD8	BUV737	53-6.7	BD	1:200	612759	RRID:AB_2870090
CD4	BUV395	RM4-5	BD	1:200	740208	RRID:AB_2734761
MHCII	APC	M5/114.15.2	Biolegend	1:200	107614	RRID:AB_313329
CD80	BV605	16-10A1	Biolegend	1:100	104729	RRID:AB_11126141
CD86	BV605	GL-1	Biolegend	1:100	105037	RRID:AB_11204429
F4/80	BV421	BM8	Biolegend	1:100	123132	RRID:AB_2563102
Fixable Viability Dye	eFluor® 506	na	eBioscience	1:250	65-0866-14	Not authenticated by RRID
CD16/CD32 (Mouse Fc Block)			BD	1:200	553141	RRID:AB_394656
Normal Rat Serum			eBioscience	1:100	24-5555-93	RRID:AB_2644748
I-Ad | chicken ova 323-339	PE	ISQAVHAAH AEINEAGR	NIH Tetramer Core Facility	1:50	42747	
**Human**						
CD45	FITC	HI30	Biolegend	1:100	304038	RRID:AB_2562050
CD3	BV785	OKT3	Biolegend	1:200	317330	RRID:AB_2563507
CD4	APC-Cy7	RPA-T4	Biolegend	1:400	300518	RRID:AB_314086
CD8a	BV605	RPA-T8	Biolegend	1:400	301040	RRID:AB_2563185
CD25	BV421	BC96	Biolegend	1:200	302630	RRID:AB_11126749
CD127	BV650	A019D5	Biolegend	1:300	351326	RRID:AB_2562095
CD73	PE	AD2	Biolegend	1:200	344004	RRID:AB_2298698
CD39	PE-Cy7	A1	Biolegend	1:200	328212	RRID:AB_2099950
