# Supplementary material for: Pregnancy-acquired memory CD4+ regulatory T cells improve pregnancy outcome in mice
Source: Nat Commun. 2025 Jul 15;16:6522. doi: 10.1038/s41467-025-61572-w (PMC12263984; doi:10.1038/s41467-025-61572-w)
Supplement: Supplementary file 2 — Reporting Summary [file 41467_2025_61572_MOESM2_ESM.pdf]

nature portfolio

Corresponding author(s): Kristin Thiele

Last updated by author(s): 15.05.2025

Reporting Summary

Nature Portfolio wishes to improve the reproducibility of the work that we publish. This form provides structure for consistency and transparency in reporting. For further information on Nature Portfolio policies, see our Editorial Policies and the Editorial Policy Checklist.

Statistics

For all statistical analyses, confirm that the following items are present in the figure legend, table legend, main text, or Methods section.

n/a	Confirmed	
☐	☒	The exact sample size (*n*) for each experimental group/condition, given as a discrete number and unit of measurement
☐	☒	A statement on whether measurements were taken from distinct samples or whether the same sample was measured repeatedly
☐	☒	The statistical test(s) used AND whether they are one- or two-sided<br>*Only common tests should be described solely by name; describe more complex techniques in the Methods section.*
☒	☐	A description of all covariates tested
☒	☐	A description of any assumptions or corrections, such as tests of normality and adjustment for multiple comparisons
☐	☒	A full description of the statistical parameters including central tendency (e.g. means) or other basic estimates (e.g. regression coefficient) AND variation (e.g. standard deviation) or associated estimates of uncertainty (e.g. confidence intervals)
☐	☒	For null hypothesis testing, the test statistic (e.g. *F*, *t*, *r*) with confidence intervals, effect sizes, degrees of freedom and *P* value noted<br>*Give P values as exact values whenever suitable.*
☒	☐	For Bayesian analysis, information on the choice of priors and Markov chain Monte Carlo settings
☒	☐	For hierarchical and complex designs, identification of the appropriate level for tests and full reporting of outcomes
☐	☒	Estimates of effect sizes (e.g. Cohen's *d*, Pearson's *r*), indicating how they were calculated

Our web collection on statistics for biologists contains articles on many of the points above.

Software and code

Policy information about availability of computer code

Data collection	BD FACS DIVA, Becton Dickinson
Data analysis	FlowJo, vX. FLowJo LLC.; Prism v.9, GraphPad; R package InfinityFlow (v1.6.0) with XGBoost

For manuscripts utilizing custom algorithms or software that are central to the research but not yet described in published literature, software must be made available to editors and reviewers. We strongly encourage code deposition in a community repository (e.g. GitHub). See the Nature Portfolio guidelines for submitting code & software for further information.

Data

Policy information about availability of data

All manuscripts must include a data availability statement. This statement should provide the following information, where applicable:

- Accession codes, unique identifiers, or web links for publicly available datasets
- A description of any restrictions on data availability
- For clinical datasets or third party data, please ensure that the statement adheres to our policy

Source data are provided with this paper. Flow cytometry data were made publically available under FlowRepository Access Code: FR-FCM-Z85N. Further information and resources and reagents are available from the corresponding author on request.

Research involving human participants, their data, or biological material

Policy information about studies with human participants or human data. See also policy information about sex, gender (identity/presentation), and sexual orientation and race, ethnicity and racism.

Reporting on sex and gender	The PRINCE study is a prospective longitudinal pregnancy cohort study. Hence, recruitmemt was restricted to pregnant women. A distinction in terms of sex was only made in the children.
Reporting on race, ethnicity, or other socially relevant groupings	Nationality and ethnic origin were surveyed by means of a questionnaire. As the recruitment took place at the University Medical Center Hamburg-Eppendorf, the majority of participants were Central European.
Population characteristics	All pregnant women were above the age of 18 years or higher and had a viable singleton pregnancy at gestational week 12–14. The following exclusion criteria were defined: women with chronic infections (HIV, hepatitis B/C), known substance abuse and smoking, multiple pregnancies or pregnancies conceived after assisted reproductive technologies. Assessment of relevant covariables has been described in detail in Diemert et al., J Reprod Immunol 2017, PMID: 28641119.
Recruitment	Pregnant women were recruited in their first trimester (12.-14. weeks of gestation).
Ethics oversight	All study subjects signed informed consent forms and the study protocol was approved by the ethics committee of the Hamburg Chamber of Physicians (PV3694).

Note that full information on the approval of the study protocol must also be provided in the manuscript.

Field-specific reporting

Please select the one below that is the best fit for your research. If you are not sure, read the appropriate sections before making your selection.

☒ Life sciences ☐ Behavioural & social sciences ☐ Ecological, evolutionary & environmental sciences

For a reference copy of the document with all sections, see nature.com/documents/nr-reporting-summary-flat.pdf

Life sciences study design

All studies must disclose on these points even when the disclosure is negative.

Sample size	An a priori power analysis was conducted using G*Power v3.1 software (Heinrich Heine Universität Düsseldorf). We mostly aimed for 5-8 mice per group depending on the variables used.<br>Sample size of the human cohort was determined based on the number of participants who participated with two consecutive pregnancies in the PRINCE study. We excluded participants who participated twice, but first pregnancy in the study did not equal first pregnancy of the woman.
Data exclusions	Outlier were excluded based on GraphPad Prism ROUT outlier analysis, Q = 10%.
Replication	Most of the experiments were independently repeated at at least two different gestational days or two different mouse lines (stress experiments) to verify the findings, which were successful.
Randomization	Groups of female mice were age-matched and randomly asigned to be mated once or twice, respectively. For any interventions during gestation, mice were also randomly split to control and treatment group.
Blinding	Investigators were not blinded during the experiments or analysis of the data.

Reporting for specific materials, systems and methods

We require information from authors about some types of materials, experimental systems and methods used in many studies. Here, indicate whether each material, system or method listed is relevant to your study. If you are not sure if a list item applies to your research, read the appropriate section before selecting a response.

Materials & experimental systems

n/a	Involved in the study
☐	☒ Antibodies
☒	☐ Eukaryotic cell lines
☒	☐ Palaeontology and archaeology
☐	☒ Animals and other organisms
☐	☒ Clinical data
☒	☐ Dual use research of concern
☒	☐ Plants

Methods

n/a	Involved in the study
☒	☐ ChIP-seq
☐	☒ Flow cytometry
☒	☐ MRI-based neuroimaging

Antibodies

Antibodies used

Antigen/Target Conjugated fluorochrome Clone Company Dilution Catalog number IDENTIFIER

Mouse
CD45 APC-Cy7 30-F11 BD 1:400 422302 RRID: AB_2818986
CD3 PE-Cy7 145-2C11 Biolegend 1:200 100319 RRID:AB_312684
CD3 PE eFluor® 610 145-2C11 eBioscience 1:100 61-0031-80 RRID:AB_2574513
CD8 BV650 53-6.7 Biolegend 1:100 100741 RRID:AB_11124344
CD4 BV605 RM4-5 Biolegend 1:200 100547 RRID:AB_2563054
CD4 AF700 GK1.5 Biolegend 1:200 100430 RRID:AB_493699
CD25 APC PC61 Biolegend 1:200 102011 RRID:AB_312860
CD25 BV605 PC61 Biolegend 1:200 102035 RRID:AB_11126977
CD44 PE IM7 BD 1:100 553134 RRID:AB_394649
CD44 Pacific blue IM7 Biolegend 1:100 103020 RRID:AB_493683
CD62L BV711 MEL-14 Biolegend 1:200 104445 RRID:AB_2564215
FoxP3 FITC FJK-16s eBioscience 1:100 11-5773-80 RRID:AB_465242
FoxP3 PE FJK-16s eBioscience 1:100 12-5773-80 RRID: AB_465935
CD69 PE-Cy7 H1.2F3 BD 1:100 552879 RRID:AB_394508
CD103 Pacific Blue 2F7 Biolegend 1:100 121418 RRID:AB_2128619
CD11c BV785 N418 Biolegend 1:100 117335 RRID:AB_2565268
CD11b PE-Cy7 M1/70 Biolegend 1:400 101216 RRID:AB_312799
CD8 BUV737 53-6.7 BD 1:200 612759 RRID:AB_2870090
CD4 BUV395 RM4-5 BD 1:200 740208 RRID:AB_2734761
MHCII APC M5/114.15.2 Biolegend 1:200 107614 RRID:AB_313329
CD80 BV605 16-10A1 Biolegend 1:100 104729 RRID:AB_11126141
CD86 BV605 GL-1 Biolegend 1:100 105037 RRID:AB_11204429
F4/80 BV421 BM8 Biolegend 1:100 123132 RRID:AB_2563102
Fixable Viability Dye eFluor® 506 na eBioscience 1:250 65-0866-14 Not authenticated by RRID
CD16/CD32 (Mouse Fc Block) BD 1:200 553141 RRID:AB_394656
Normal Rat Serum eBioscience 1:100 24-5555-93 RRID:AB_2644748
I-Ad | chicken ova 323-339 PE ISQAVHAAHAEINEAGR NIH Tetramer Core Facility 1:50 42747

Human
CD45 FITC HI30 Biolegend 1:100 304038 RRID:AB_2562050
CD3 BV785 OKT3 Biolegend 1:200 317330 RRID:AB_2563507
CD4 APC-Cy7 RPA-T4 Biolegend 1:400 300518 RRID:AB_314086
CD8a BV605 RPA-T8 Biolegend 1:400 301040 RRID:AB_2563185
CD25 BV421 BC96 Biolegend 1:200 302630 RRID:AB_11126749
CD127 BV650 A019D5 Biolegend 1:300 351326 RRID:AB_2562095
CD73 PE AD2 Biolegend 1:200 344004 RRID:AB_2298698
CD39 PE-Cy7 A1 Biolegend 1:200 328212 RRID:AB_2099950

Validation

All antibodies were validated by the respective manufacturer, as indicated in the respective data sheets.
BioLegend performs quality control for all antibodies, focusing on verifying specificity, purity, and stability. They utilize various methods, including immunofluorescent staining with flow cytometric analysis and Western blotting, to ensure consistent lot-to-lot performance. BD Biosciences antibodies adhere to strict quality control SOPs and guidelines to ensure lot-to-lot consistency. This includes quality control testing of new, manufactured lots are performed side-by-side with a previously accepted lot as a control, helping to serve as a reference for comparison and assuring that performance of the new lot is both reliable and consistent. For the antibody against FoxP3, supplied by eBioscience, the antibody is routinely quality-control tested for intracellular staining (flow cytometry) by the supplier.
For quality control, we titrate each antibody to determine its dilution for optimal performance for each application, and repeat its titration after receipt of each new lot.

Animals and other research organisms

Policy information about studies involving animals; ARRIVE guidelines recommended for reporting animal research, and Sex and Gender in Research

Laboratory animals	Female Fir/Tiger, DEREG and Fate+ mice (all C57Bl/6J background) as well as male Balb/c or OVA-Balb/c mice were obtained from the animal breeding facility of University Medical Center Hamburg-Eppendorf. Female C57BL/6J and male DBA mice were purchased from Charles River.<br>Mice were single-housed (males) or maintained in groups (females) in the animal facility of University Medical Center Hamburg-Eppendorf with regular chow and water provided ad libitum in a normal 12-hour light/12-hour dark cycle at a room temperature of 21°C and controlled humidity at 43%. Experiments were performed using age-matched 8-10-week-old females. Males were used for mating from fertile age up until 1 year of age.
Wild animals	Study did not involve wild animals.
Reporting on sex	All experimental animals were females, since all experiments were dealing with pregnancy. Assessment of fetal outcome (e.g. fetal weight) was not stratified by sex. However, fetal tails were collected in some experiments to assess the sex retrospectively, if requested.
Field-collected samples	Study did not involve samples collected in the field.
Ethics oversight	Animal care and all experimental procedures were performed according to University Medical Center Hamburg-Eppendorf institutional guidelines and conform to requirements of the German Animal Welfare Act. Ethical approvals were obtained from the State Authority of Hamburg (Behörde für Gesundheit und Verbraucherschutz, Hansestadt Hamburg, Germany, approval numbers: G16/012, G17/049, N20/14 and ORG_1009.

Note that full information on the approval of the study protocol must also be provided in the manuscript.

Clinical data

Policy information about clinical studies

All manuscripts should comply with the ICMJE guidelines for publication of clinical research and a completed CONSORT checklist must be included with all submissions.

Clinical trial registration	Ethics committee of the Hamburg Chamber of Physicians (PV3694).
Study protocol	Assessment of relevant covariables has been described in detail in Diemert et al., J Reprod Immunol 2017, PMID: 28641119.
Data collection	The PRINCE study is a population-based prospective pregnancy study based at the University Medical Center Hamburg-Eppendorf and was initiated in 2011. Pregnant women were invited to three antenatal visits, once per trimester (gestational weeks 12 to 14, 24 to 26, and 34 to 36). During these visits participants were ask to fill out a detailed questionaire. Further, a detailed ultrasound was performed and blood sample were taken to isolate PBMCs and serum samples.
Outcomes	Data on personal information, previous pregnancies, socio-demographics and living situation, previous illnesses, e.g. allergies. Further, clinical data were assessed via ultrasound. Information on medication, food consumption and emotional and psychological well-being, including social support were documented.

Plants

Seed stocks	The manuscript does NOT include experiments with plants.
Novel plant genotypes	*Describe the methods by which all novel plant genotypes were produced. This includes those generated by transgenic approaches, gene editing, chemical/radiation-based mutagenesis and hybridization. For transgenic lines, describe the transformation method, the number of independent lines analyzed and the generation upon which experiments were performed. For gene-edited lines, describe the editor used, the endogenous sequence targeted for editing, the targeting guide RNA sequence (if applicable) and how the editor was applied.*
Authentication	*Describe any authentication procedures for each seed stock used or novel genotype generated. Describe any experiments used to assess the effect of a mutation and, where applicable, how potential secondary effects (e.g. second site T-DNA insertions, mosiacism, off-target gene editing) were examined.*

Flow Cytometry

Plots

Confirm that:

☒ The axis labels state the marker and fluorochrome used (e.g. CD4-FITC).

☒ The axis scales are clearly visible. Include numbers along axes only for bottom left plot of group (a 'group' is an analysis of identical markers).

☒ All plots are contour plots with outliers or pseudocolor plots.

☒ A numerical value for number of cells or percentage (with statistics) is provided.

Methodology

Sample preparation	Lymph nodes were mechanically disrupted and passed through a cell strainer. The uterus was enzymatically digested using 200 U/mL hyaluronidase (Sigma-Aldrich), 1 mg/mL collagenase VIII type (Sigma-Aldrich), and 1 mg/mL bovine serum albumin fraction V (Sigma-Aldrich) dissolved in 5 mL HBSS. Solution was incubated twice for 20 minutes in a 37°C water bath with agitation and intermediately and subsequently recovered and filtered through a cell strainer. Finally, lymph node and uterine cell suspensions were centrifugated at 450 g for 8 minutes at 4°C and obtained cell pellets were resuspended in PBS. PBMCs were isolated from blood samples by using 1x Red Blood Cell (RBC) lysis buffer (eBioscience, Invitrogen by Thermo Fisher Scientific) according to the manufacturer's instructions. Lysis was stopped with PBS and subsequently, samples were centrifuged at 450 x g for 8 minutes at 4°C and resuspended PBS.
Instrument	BD Fortessa II and BD Symphony A3
Software	FACS DIVA, BD; FlowJo, FlowJo, LLC.
Cell population abundance	Purity of sorted populations was determined to be above 90%.
Gating strategy	Doublets were then excluded using (1) FSC-A vs. FSC-H, and (2) SSC-A vs. SSC-H. Subsequently viable (Fixable Viability Dye-negative) CD45 positive cells were selected to set a living, singlet leukocyte population as basis for downstream gating analyses as specified in the supplementary materials, in short: Living CD45 --> CD3 --> CD4 --> FoxP3 to analysis CD4 regulatory T cells. If necessary, gate boundaries between positive and negative cell subsets were determined using fluorescence-minus-one (FMO) control stainings.

☒ Tick this box to confirm that a figure exemplifying the gating strategy is provided in the Supplementary Information.
